# Consistency Test between Scoring Systems for Predicting Outcomes of Chronic Myeloid Leukemia in a Saudi Population Treated with Imatinib

**DOI:** 10.1155/2017/1076493

**Published:** 2017-02-13

**Authors:** Haneen R. Banjar, Enaam Alsobhi

**Affiliations:** ^1^The Department of Computer Science, King Abdulaziz University, Jeddah, Saudi Arabia; ^2^School of Computer Science, University of Adelaide, Adelaide, SA, Australia; ^3^Pathology and Laboratory Medicine, King Abdulaziz Medical City, National Guard Health Affairs, Jeddah, Saudi Arabia

## Abstract

Inconsistency in prognostic scores occurs where two different risk categories are applied to the same chronic myeloid leukemia (CML) patient. This study evaluated common scoring systems for identifying risk groups based on patients' molecular responses to select the best prognostic score when conflict prognoses are obtained from patient profiles. We analyzed 104 patients diagnosed with CML and treated at King Abdulaziz Medical City, Saudi Arabia, who were monitored for major molecular response (achieving a* BCR-ABL1* transcript level equal to or less than 0.1%) by Real-Time Quantitative Polymerase Chain Reaction (RQ-PCR), and their risk profiles were identified using Sokal, Hasford, EUTOS, and ELTS scores based on the patients' clinical and hematological parameters at diagnosis. Our results found that the Hasford score outperformed other scores in identifying risk categories for conflict groups, with an accuracy of 63%.

## 1. Introduction

The Australian Institute of Health and Welfare (AIHW) classified myeloid cancers as the ninth most commonly diagnosed cancer in 2016, with more than 3,600 cases in Australia [1]. Chronic myeloid leukemia (CML) is also known as chronic myelogenous leukemia or chronic granulocytic leukemia. The bone marrow produces an unusual number of white blood cells. The bone marrow could produce an excessive number of immature white blood cells and lead to progressive disease. Consequently, the bone marrow cannot make enough red cells, normal white cells, and platelets [2].

Prognostic scores in patients with CML are used to stratify CML patients according to risk profile to ensure appropriate treatment. Historically, the science of prognostication has evolved rapidly, and various scoring systems have been developed to optimize the use of clinical experience in CML treatment. These scores were developed using logistic regression with the selection of the patients' clinical and hematological parameters at diagnosis. The common prognostic scores have shown variable correlation with complete cytogenetic response (CCyR) [3–8] and major molecular response (MMR) [9–12]. Although the investigation compared the prognostic value of the validated scoring systems in overall survival (OS), event free survival (EFS) or optimal response in CML patients who receive frontline imatinib, applying the established prognostic scores in a comparative fashion and questioning the value of scoring systems, especially with regard to inconsistency in risk category, has not been considered in previous studies.

The European LeukemiaNet (ELN) current recommendations for the management of CML are basically addressed to the goal of achieving an at least MMR [13]. As newly diagnosed CML patients should be stratified based on the available prognostic scoring systems, we considered the risk groups might be studied based on the MMR outcomes. This is needed to evaluate the clinical impact of the existing prognostic scores by comparison of prognostic risk groups with primary concern on consistency in prognostic scores outcomes. Inconsistency occurs when two different risk categories are applied to the same CML patient; that is, one prognostic score classifies the patient in one group and the other score contradicts the first classification. Consistency in prognostic scores used to estimate the risk group of CML patients before therapy commencement can increase clinician trust in the treatment decision and play important role in modern medicine for CML changing treatment modalities [14, 15]. However, conflict between prognostic scores is observed in some CML patients. Thus, it is important to study consistency between prognostic score categories used to allocate CML patients to risk groups in order to support clinician decision-making.

Our analysis evaluated the different scores outcomes with the long-term molecular response in patients treated with imatinib to determine which was the best prognostic score to apply where a conflict prognosis was generated by prognostic scores.

## 2. Materials and Methods

### 2.1. Study Population

Participants in this study were members of the Saudi population diagnosed with CML and treated at King Abdulaziz Medical City, Jeddah [16]. A total of 104 CML patients received 400 mg imatinib as the initial therapy. Patient characteristics are described in Table 1. All of the patients monitored their MMR in time points defined by ELN [13] where MMR is defined as achieving a* BCR-ABL1* transcript level equal to or less than 0.1% at 12 months by RQ-PCR.

### 2.2. Scoring Systems in CML

Four common prognostic scoring systems are available for CML patients prior to commencing therapy: (1) the Sokal score [17], (2) the Hasford score [14], (3) the European Treatment and Outcome Study (EUTOS) score [15], and (4) the EUTOS long-term survival (ELTS) score [18]. These four scores ascertain the level of risk for CML patients by running multivariable regression analysis. Prognostic scores were calculated using formulas in Table 2, based on the patients' clinical and hematological parameters at diagnosis.

The analysis is conducted in two steps: (1) studying the prognostic index using combined groups and (2) consistency analysis between the risk categories obtained from the scoring systems. First, from Table 2, the EUTOS score is the only score that classifies CML patients into low risk and high risk. The number of categories in comparative prognostic scores in Sokal, Hasford, EUTOS, and ELTS was three, three, two, and three, respectively. Accuracy was measured on prognostic score data by assuming two different combined groups: (1) low and intermediate risk in Sokal, Hasford, and ELTS scores as low risk and (2) intermediate and high risk in Sokal, Hasford, and ELTS scores as high risk.

Secondly, in consistency analysis, the combined category is selected based on the higher-accuracy results from combined groups to study the inconsistency between scoring systems. We are dealing with two models advising on the same patient. Each score may provide an index that conflicts with the other. The patients were classified into a consistency group or an inconsistency group. The consistency group included patients who observed consistent risk categorization from scoring systems, while the inconsistency group included patients who observed inconsistent risk categorization from scoring systems. The possible combination of risk categories for *S* scoring systems is *N* (number of the risk categories) raised to *S* power. The number of patients belongs to each molecular response groups is included to calculate the accuracy and determine which is the most accurate scoring system that can be used in a conflict group.

## 3. Results and Discussion

This study presents the analysis of each scoring system for distinguishing patients. We evaluated scoring systems in CML for identifying risk categories based on patients' molecular responses to determine which was the best prognostic score to apply where a conflict prognosis was generated by prognostic scores.

Of the 104 CML patients included in this study, the data of 9 patients were removed due to incomplete MMR data, to improve overall data quality. Of the 95 patients with complete data, 33 (34%) did not achieve MMR, while 62 (65%) did achieve MMR. The number of CML patients per prognostic score included in the two different combined methods is shown in Table 3.

It is clearly observed that the combined method of low and intermediate risk in Sokal, Hasford, and ELTS score as low risk achieved higher accuracy than the second combined method of intermediate and high risk in Sokal, Hasford, and ELTS score as high risk. Comparison of the accuracies in Sokal was 62.10% versus 48.42%, Hasford was 67.37% versus 58.94%, and ELTS was 62.10% versus 61.05%. Indeed, the ELN [13] recommended dividing patients into low-risk (including intermediate) and high-risk populations in the management of CML. Basically, there is insufficient evidence to prove intermediate risk patients behave differently from low-risk patients. A study used the combined method of low and intermediate in one risk group to evaluate Sokal and EUOS to predict optimal response [12]. Therefore, we used the first combined method in the consistency analysis.

In Table 4, there will be sixteen rows in our analysis (2^4^ = 16). The consensus group involved 65 (68.42%) patients, and there were 30 (31.58%) patients in the conflict group. To identify the most appropriate prognostic score to use when there is conflict between prognostic scores, we compared the number of patients belonging to each group. Table 4 shows that, in the consensus group, both prognostic scores incorrectly predict CML risk group in 21% (19 patients did not achieve MMR, while all scores classified them in the low-risk group, and 1 achieved MMR, while all scores classified this patient in the high-risk group) of cases. In the conflict group, the Sokal and ELTS scores predicted MMR accurately in 46.67% (14 of 30) of patients, while the EUTOS score predicted MMR accurately in 50% (15 of 30) of patients. The highest accuracy of 63.33% (19 of 30) of patients was obtained by the Hasford score for predicting the risk category. However, the accuracy achieved by the Hasford score in both groups (consensus and conflict groups) was the lowest (58.95%) among the other scores (Sokal's accuracy: 62.11%, EUTOS's accuracy: 63.16%, and ELTS's accuracy: 62.11%).

Although the results show that the Hasford performance in the consensus and conflict groups was not recommended, the Hasford score accuracy percentage (63%) shows that Hasford may be useful in identifying risk group in conflict CML patients. In the conflict group, the Hasford prognostic score identified more low-risk categories for CML patients and few high-risk patients, while the Sokal score identified more high-risk patients and few low-risk patients. Only one study [3] reported conflict in 22 CML patients. This study also supports our finding as they found that a majority of patients corroborated better with the Hasford score [14] than the Sokal and EUTOS scores. Previous studies compared and assessed the Sokal, Hasford, and EUTOS but not ELTS scores in investigating consistency between the scoring systems. Our study is the first to investigate the conflict and compare the four validated scoring systems. Comparison of prognostic scores shows the diversity in scoring, but in future work, we intend to implement advanced methods from computer science to resolve conflict. Thus, a new scoring system combining the power of currently available prognostic scores may further help increase accuracy of identifying risk groups.

## Figures and Tables

**Table 1:** Characteristics of 95 patients with CML at diagnosis.

Factor	Median	Range	SD
Age (yrs)	40.21	18–74	15.13
Spleen size (cm, BCM)	8.33	0–25	7.53
Platelet count (×10^9^/L)	510.97	4.42–2876	439.88
Basophils (%)	1.32	0–7	1.10
Eosinophils (%)	0.83	0–0.07	1.24
Peripheral blast (%)	1.50	0–10	1.39

SD = standard deviation; BCM = below costal margin.

**Table 2:** The current methods used to identify risk in CML.

Scoring systems						
Study	Factors	Equation	Method	Risk categories	Target prediction	Data and results
Sokal score, Sokal et al. [17]	Age, spleen size (cm), blast (%), and platelets (10^9^/L)	exp(0.0116 × (age [years] − 43.4)) + 0.0345 × (spleen size [cm] − 7.51) + (0.188 × ((platelets [10^9^/L]/700)^2^ − 0.563)) + (0.0887 × (blasts [%] − 2.10))	Multivariate analysis of survival	Low-risk score < 0.8, of patients, intermediate-risk in 0.8–1.2, and high-risk > 1.2	Risk groups for chemotherapy	Six European and American sources (*n* = 813), low 39%, intermediate 38%, and high 23%

Hasford score, Hasford et al. [14]	Age, spleen size (cm), blasts (%), eosinophils (%), basophils (%), and platelets (10^9^/L)	(0.6666 × age [0 when age < 50 years; 1 otherwise]) + (0.0420 × spleen size [cm]) + (0.0584 × blasts [%]) + (0.0413 × eosinophils [%]) + (0.2039 × basophils [0 when basophils < 3%; 1 otherwise]) + (1.0956 × platelet count [0 when platelets < 1500 × 10^9^/L; 1 otherwise]) × 1000	Multivariate analysis of survival	Low-risk score ≤ 780, intermediate-risk in 781–1480, and high-risk ≥ 1481	Risk groups for interferon alpha alone	14 studies (*n* = 981), low 40.6%, intermediate 44.7%, and high 14.6%

EUTOS score, Hasford et al. [15]	Basophils (%) and spleen size (cm)	(7 × basophil [%]) + (4 × spleen [cm])	Multivariate analysis of response	Low-risk score < 87 and high-risk ≥ 87	CCgR at 18 months to imatinib	Five national study groups (*n* = 2,060), low 79% and high 21%

ELTS score, Pfirrmann et al. [18]	Age, spleen size (cm), blast (%), and platelets (10^9^/L)	0.0025 × (age in completed years/10)^3^ + 0.0615 × spleen size below costal margin + 0.1052 × blasts in peripheral blood + 0.4104 × (platelet count/1000)^−0.5^	Multivariate analysis of response	Low-risk score ≤ 1.5680, intermediate-risk in 1.5680–2.2185, and high-risk > 2.2185	Long-term survival	(*n* = 2,205), low 61%, intermediate 27%, and high 12%

**Table 3:** The number of patients in different risk groups as per calculated scores.

	*n*	Not achieving MMR	Achieving MMR	Accuracy
	Combined groups (1)			
*Sokal score risk group*				
High	25	11	14	62.10
Low and intermediate	70	22	48	
*Hasford score risk group *				
High	6	4	2	67.37
Low and intermediate	89	29	60	
*EUTOS score risk group *				
High	10	4	6	63.15
Low	85	29	56	
*ELTS score risk group *				
High	17	7	10	62.10
Low and intermediate	78	26	52	

	Combined groups (2)			
*Sokal score risk group*				
Intermediate and high	62	23	39	48.42
Low	33	10	23	
*Hasford score risk group *				
Intermediate and high	46	20	26	58.94
Low	49	13	36	
*EUTOS score risk group *				
High	10	4	6	63.15
Low	85	29	56	
*ELTS score risk group *				
Intermediate and high	42	19	23	61.05
Low	53	14	39	

**Table 4:** The consistency/inconsistency of prognostic scores for predicting major molecular response.

Analysis groups	Score prediction				MMR		Total
	Sokal	Hasford	EUTOS	ELTS	Achieving MMR, *n* = 62	Not achieving MMR, *n* = 33	
Conflict group	Low and intermediate	Low and intermediate	Low	High	5	2	30
	Low and intermediate	Low and intermediate	High	Low and intermediate	0	0	
	Low and intermediate	Low and intermediate	High	High	0	1	
	Low and intermediate	High	Low	Low and intermediate	0	0	
	Low and intermediate	High	Low	High	0	0	
	Low and intermediate	High	High	Low and intermediate	0	0	
	Low and intermediate	High	High	High	0	0	
	High	Low and intermediate	Low	Low and intermediate	7	5	
	High	Low and intermediate	Low	High	0	1	
	High	Low and intermediate	High	Low and intermediate	1	1	
	High	Low and intermediate	High	High	4	0	
	High	High	Low	Low and intermediate	1	1	
	High	High	Low	High	0	1	
	High	High	High	Low and intermediate	0	0	

Consensus group	Low and intermediate	Low and intermediate	Low	Low and intermediate	43	19	65
	High	High	High	High	1	2	

Single score in consensus group and conflict group	Low and intermediate				48	22	95
	High				14	11	
		Low and intermediate			36	13	
		High			26	20	
			Low		56	29	
			High		6	4	
				Low and intermediate	52	26	
				High	10	7	

Single score in conflict group	Low and intermediate				5	3	30
	High				13	9	
		Low and intermediate			17	10	
		High			1	2	
			Low		13	10	
			High		5	2	
				Low and intermediate	9	7	
				High	9	5	
